# Supplementary material for: Dynamics of type IV collagen 7S fragment on eradication of HCV with direct antiviral agents: Prognostic and metabolomic impacts
Source: PLoS One. 2022 Oct 27;17(10):e0276925. doi: 10.1371/journal.pone.0276925 (PMC9612469; doi:10.1371/journal.pone.0276925)
Supplement: S1 File — (PDF) [file pone.0276925.s001.pdf]

# Supporting Information

# Supporting Figures and Legends

**S1 Fig.**

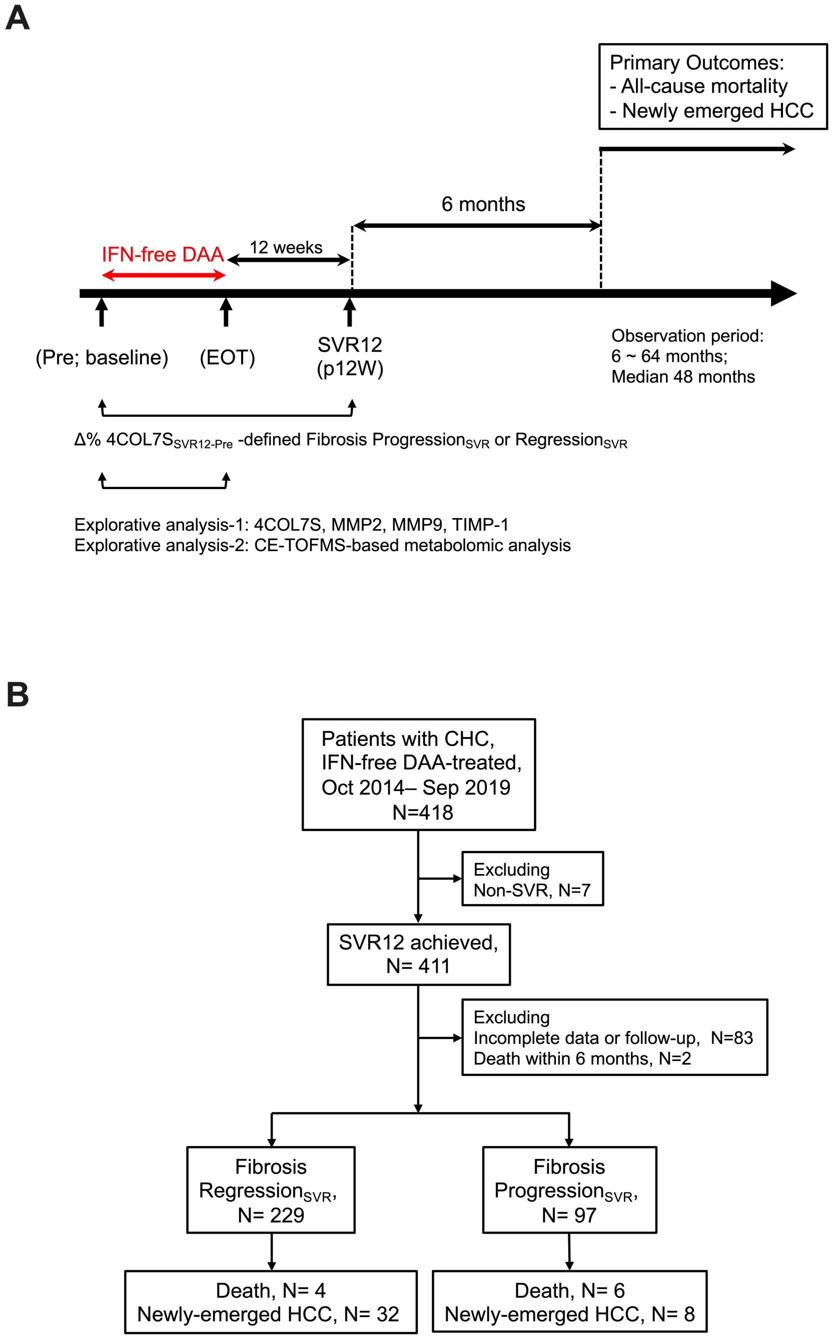


**S1 Fig**
**Yamataka et al.**

**S1 Fig. Study design, sampling timepoints, and inclusion flow.** The red arrow in panel A represents the duration of treatment with IFN-free direct antiviral agents.

Abbreviations: IFN, interferon; DAAs, direct antiviral agents; Pre, pre-treatment; EOT, end of treatment; p12w, 12 weeks after treatment; SVR12, sustained virological response at 12 weeks after the end of treatment; HCC, hepatocellular carcinoma; 4COL7S, type IV collagen 7S fragment; MMP, matrix metalloproteinase; TIMP, tissue inhibitor of metalloproteinase.

**S2 Fig.**

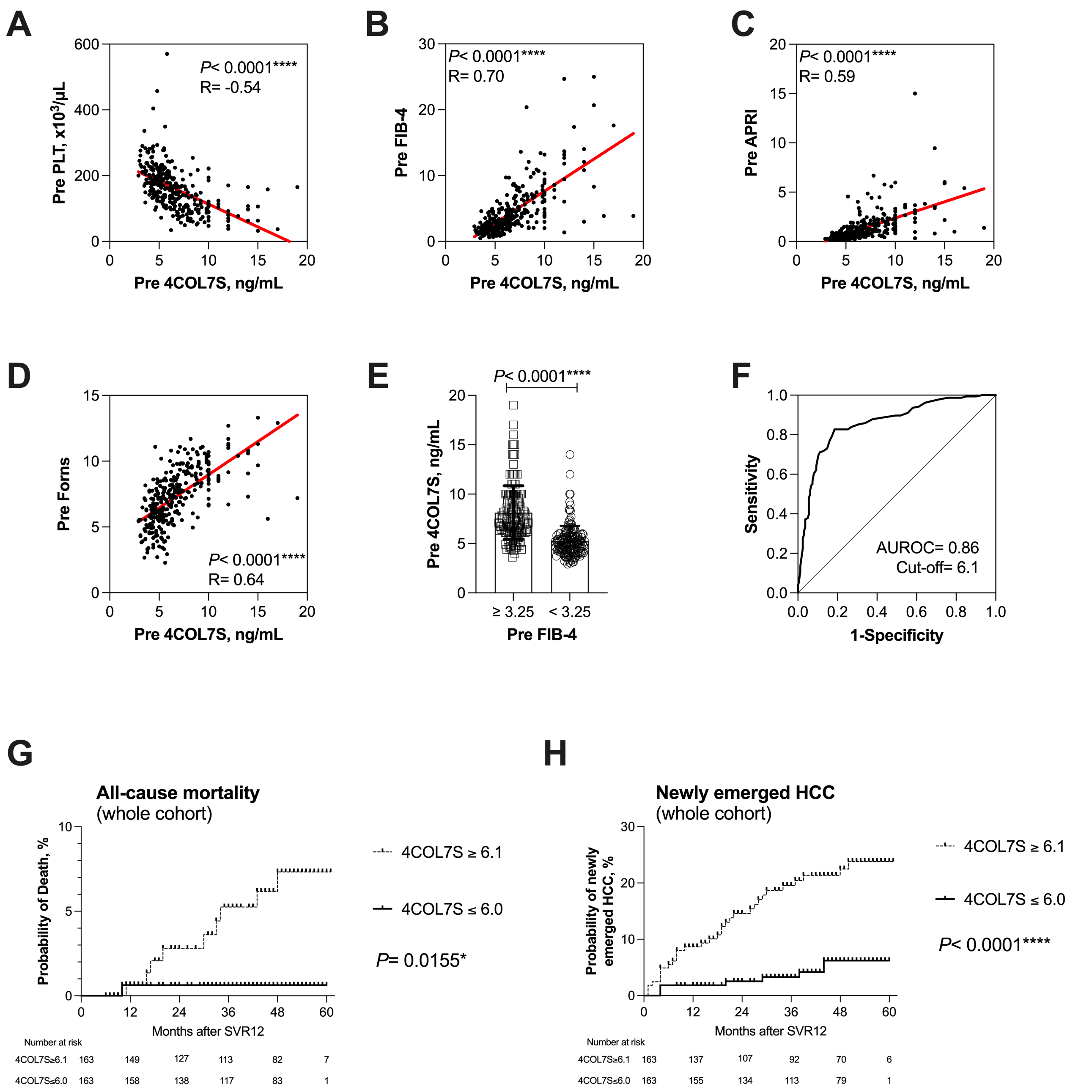


**S2 Fig. Correlations between pre-treatment 4COL7S, non-invasive fibrosis indicators and post-SVR clinical outcomes in patients with chronic hepatitis C.**

In 326 patients with CHC included in this study, correlations between pre-treatment serum levels of 4COL7S and platelet counts (A), FIB-4 (B), APRI (C), and Forns indices (D) are shown. (E) 4COL7S levels are compared based on stratifying patients with FIB-4 ≥ 3.25, which indicated advanced fibrosis. (F) An ROC analysis for serum levels of 4COL7S

diagnosing FIB-4 ≥ 3.25 is shown. A cutoff of 6.1 is yielded from the Youden index. (G, H) Kaplan-Meier analyses of all-cause mortality and new emergence of HCC post-SVR stratified by baseline 4COL7S. Data are presented as means with standard deviations. **P*<0.05; *****P* <0.0001. Abbreviations: SVR, sustained virological response; 4COL7S, type IV collagen 7S fragment; CHC, chronic hepatitis C; ROC, receiver operating characteristic; AUROC, area under the ROC curve.

**S3 Fig.**

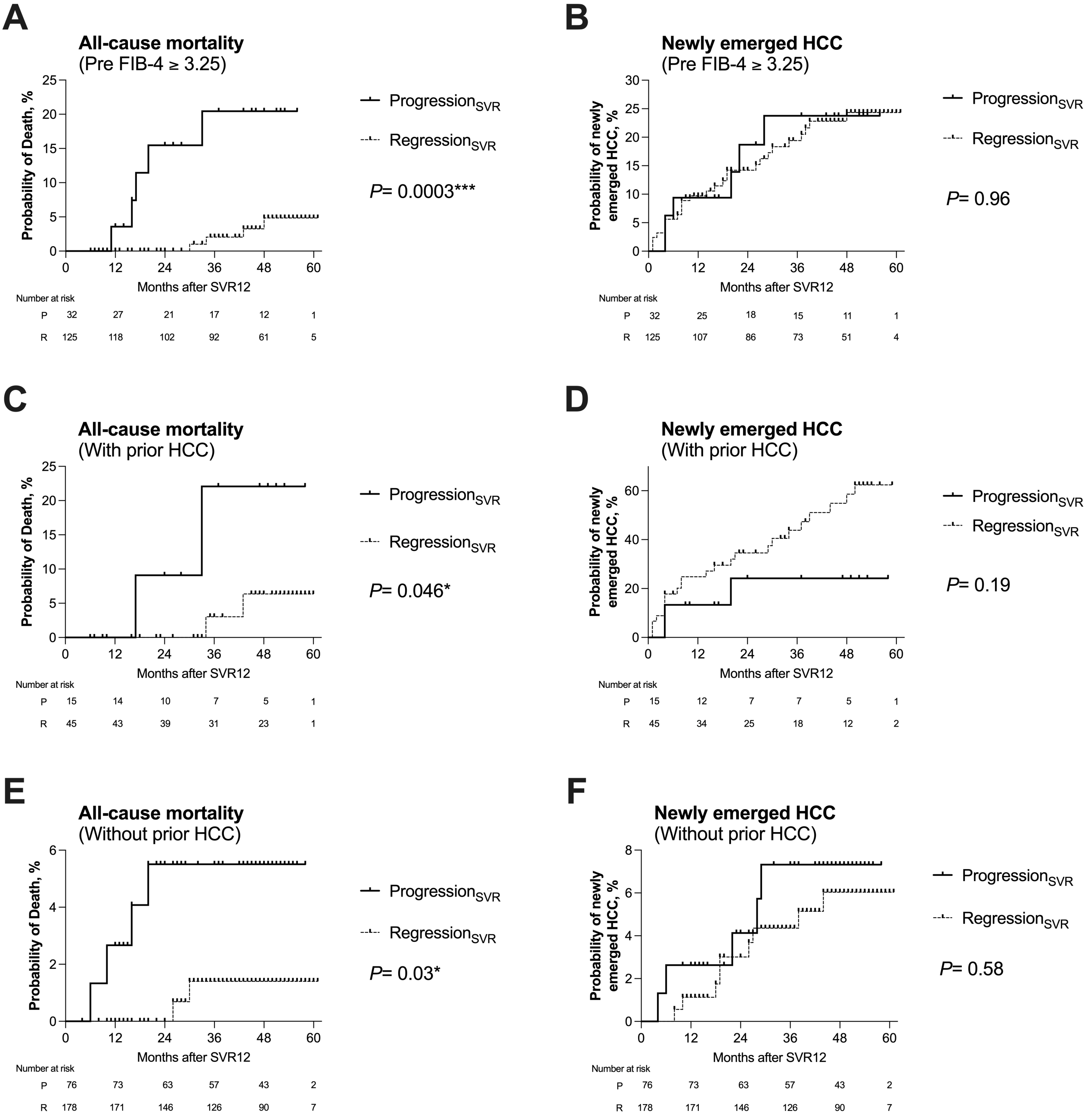


**S3 Fig. Kaplan-Meier analyses of all-cause mortality and new emergence of HCC post-SVR stratified by 4COL7S-defined fibrosis progression$_{SVR}$ and regression$_{SVR}$.**

A comparison of 4COL7S-defined fibrosis progression$_{SVR}$ and regression$_{SVR}$, all-cause mortality (A, C, E), and accumulative frequency of newly emerged HCC (B, D, F) are evaluated in SVR12 in patients whose baseline FIB-4 indices ≥ 3.25 ng/mL (N= 157; A-B) or in patients with prior HCC (n= 60, C-D) or in those without (n= 254, E-F). *$P$ < 0.05; ***$P$

<0.001. Abbreviations: 4COL7S, type IV collagen 7S fragment; P, progression$_{SVR}$; R, regression$_{SVR}$; SVR12, sustained virological response at 12 weeks after the end of treatment; HCC, hepatocellular carcinoma

**S4 Fig.**

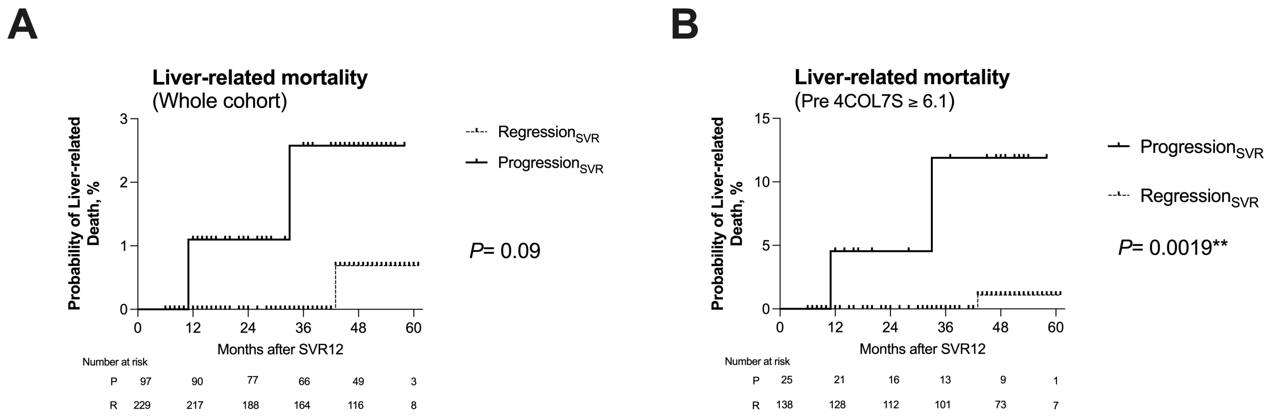


**S4 Fig. Kaplan-Meier analyses of liver-related mortality post-SVR stratified based on 4COL7S-defined fibrosis progression$_{SVR}$ and regression$_{SVR}$.**

A comparison of 4COL7S-defined fibrosis progression$_{SVR}$ and regression$_{SVR}$, liver-related mortality is evaluated from SVR12 in 326 IFN-free DAA-treated patients achieving SVR (whole cohort, A) or in those with baseline serum levels of 4COL7S ≥ 6.1 ng/mL (N= 163, B). ***P* < 0.01. Abbreviations: 4COL7S, type IV collagen 7S fragment; P, progression$_{SVR}$; R, regression$_{SVR}$; SVR12, sustained virological response at 12 weeks after the end of treatment; IFN, interferon; DAAs, direct antiviral agents.

**S5 Fig.**

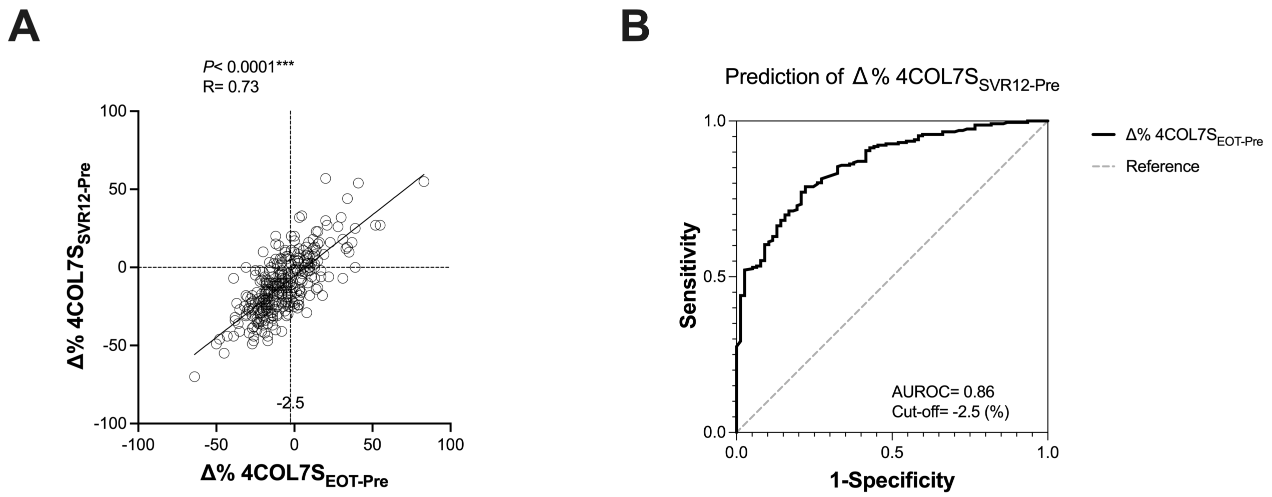


**S5 Fig. Correlations and diagnostic ability between the change rates of 4COL7S at EOT and at SVR12.**

In 326 IFN-free DAA-treated patients achieving SVR, the Spearman correlation analysis between the change rates of serum 4COL7S at EOT (Δ% $4COL7S_{EOT\text{-}Pre}$) and at SVR12 (Δ% $4COL7S_{SVR12\text{-}Pre}$) is shown (panel A). The diagnostic ability of Δ% $4COL7S_{EOT\text{-}Pre}$ for Δ% $4COL7S_{SVR12\text{-}Pre}$)≥0 by ROC analysis is shown (panel B). A cutoff of − 2.5% is obtained from the Youden index. Abbreviations: 4COL7S, type IV collagen 7S fragment; EOT, end of treatment; SVR12, sustained virological response at 12 weeks after the end of treatment; IFN, interferon; DAAs, direct antiviral agents; ROC, receiver operating characteristic

**S6 Fig.**

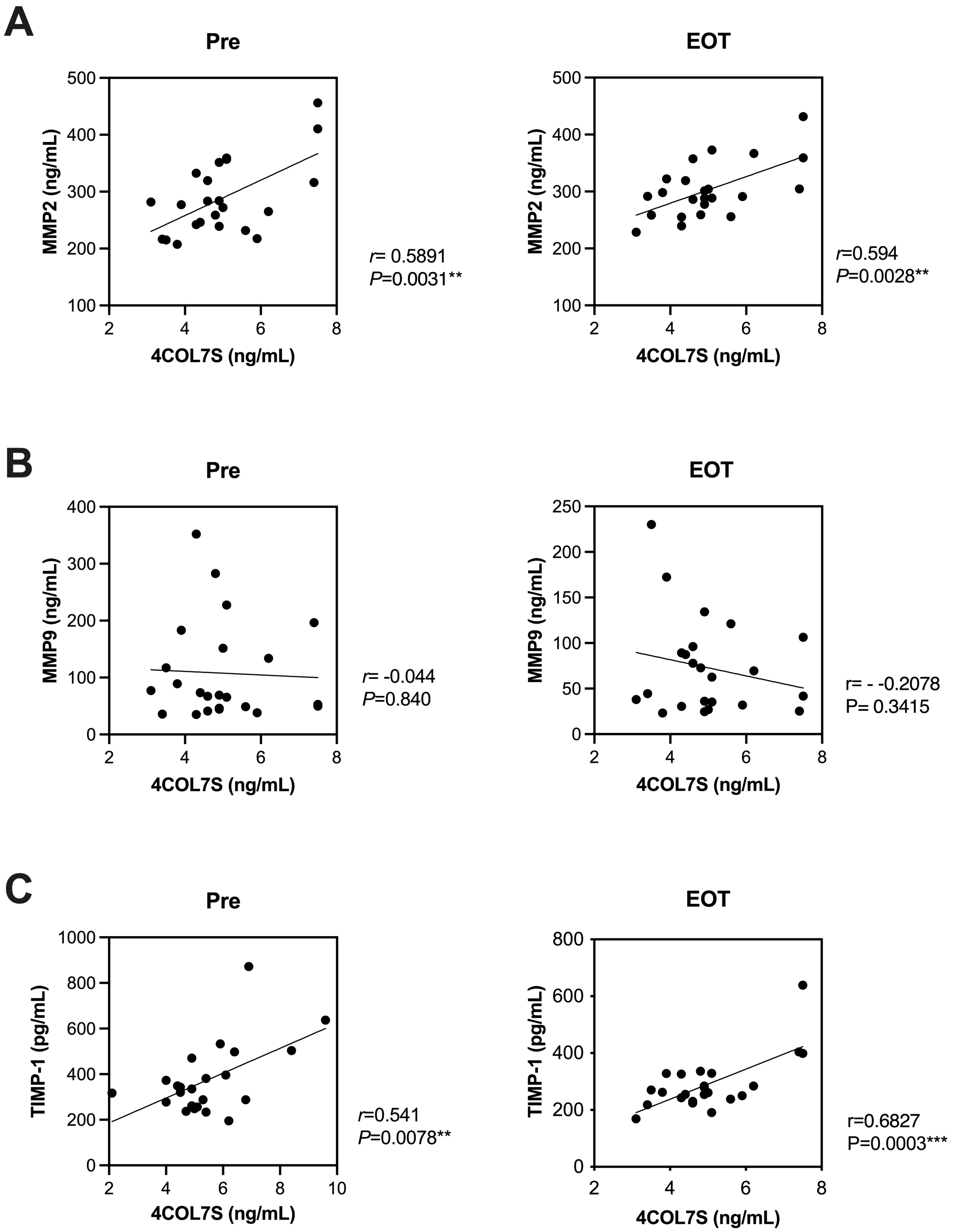


**S6 Fig. Correlations between serum 4COL7S and MMP2, MMP9, and TIMP-1 before treatment and at EOT**

In the exploratory analysis involving 23 IFN-free DAA-treated patients achieving SVR, correlations between serum 4COL7S and (A) MMP2, (B) MMP9, or (C) TIMP-1, at pre and

EOT, respectively, are shown. **$P < 0.01$; ***$P < 0.001$. Abbreviations: 4COL7S, type IV collagen 7S fragment; EOT, end of treatment; IFN, interferon; DAAs, direct antiviral agents; Pre, pre-treatment.

**S7 Fig.**

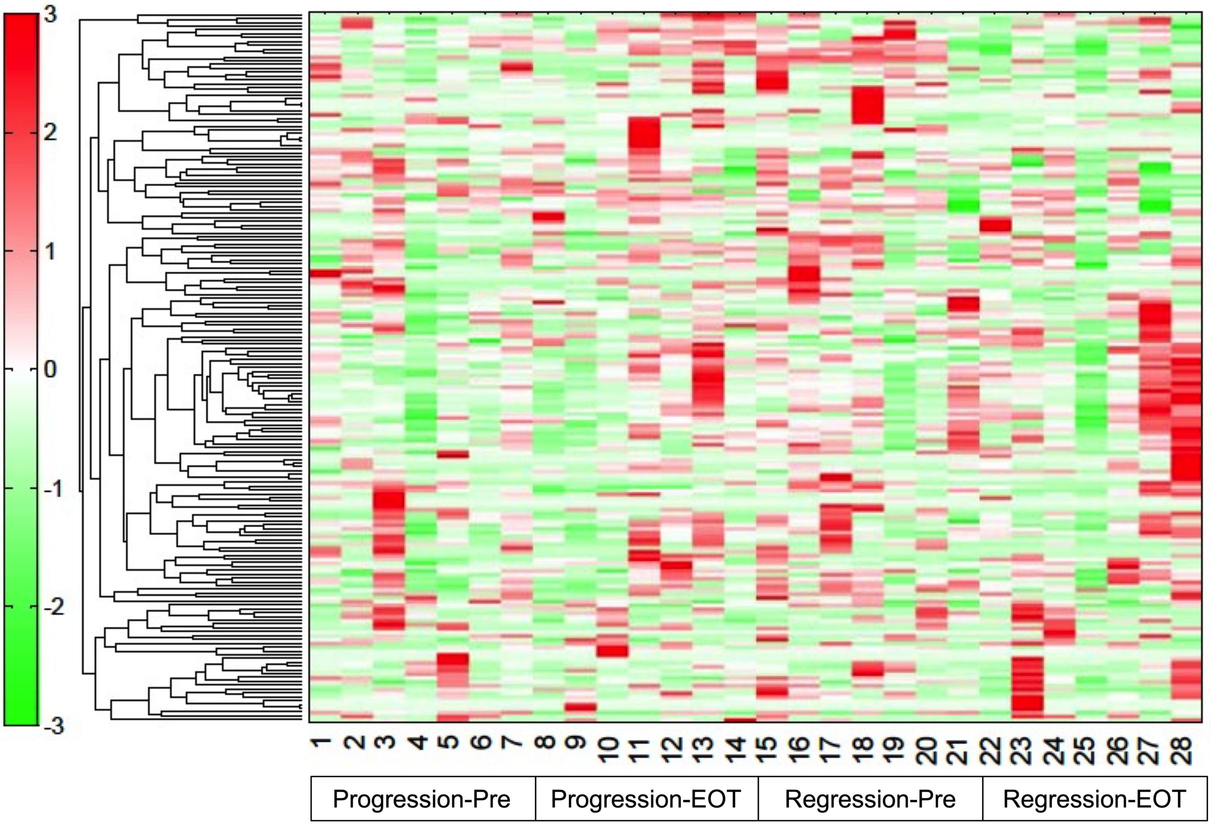


**S7 Fig. Hierarchical cluster analysis of metabolic patterns in exploratory analysis 2.**

Abbreviations: Pre, pre-treatment; EOT, end of treatment.

# Supporting Tables S1-S4

## S1 Table. Clinical features of censored deaths during observation

| | Sex | Age | Months to death (from SVR12) | Prior HCC | Baseline Platelet, $\times 10^4$/μL | Pre 4COL7S, ng/mL | SVR12 4COL7S, ng/mL | 4COL7S-defined P/R | Causes of death |
|---|---|---|---|---|---|---|---|---|---|
| 1 | M | 91 | 34 | Y | 10.3 | 6.1 | 5.6 | R | Cholangiocarcinoma |
| 2 | F | 81 | 17 | Y | 6.7 | 6.9 | 7.5 | P | Malignant Myeloma |
| 3 | M | 53 | 11 | N | 9.0 | 8.3 | 7.5 | P | HCC |
| 4 | M | 57 | 30 | N | 7.4 | 10 | 8.9 | R | CPA |
| 5 | F | 79 | 20 | N | 13.9 | 6.3 | 7.1 | P | Gastric cancer |
| 6 | F | 79 | 43 | Y | 8.0 | 12 | 11 | R | HCC |
| 7 | M | 65 | 33 | Y | 6.4 | 7.9 | 12 | P | HCC |
| 8 | F | 78 | 48 | N | 9.2 | 8 | 8.4 | R | Thyroid cancer |
| 9 | F | 78 | 16 | N | 8.9 | 10 | 11 | P | Interstitial pneumonitis |
| 10 | M | 81 | 10 | N | 21.1 | 3.3 | 3.4 | P | CPA |

Abbreviations: M, male; F, female; SVR12, sustained virological response at week 12 after end of treatment; Y, yes; N, no; HCC, hepatocellular carcinoma; 4COL7S, type IV collagen 7S fragment; P, progression; R, regression; CPA, cardiopulmonary arrest.

## S2 Table. Background clinical parameters stratified based on their fibrosis dynamics.

| Baseline parameters | Regression$_{SVR}$† | Progression$_{SVR}$† | P |
|---|---|---|---|
| N | 229 | 97 | - |
| Sex (M/F), N (%) | 82 (35.8) / 147 (64.2) | 36 (37.1) / 61 (62.9) | 0.90 |
| Age, years | 70.4± 12.0 | 70.3± 12.2 | 0.92 |
| SOF-based DAAs, N (%) | 147 (64.2) | 62 (63.9) | 1.00 |
| Prior history of HCC, N (%) | 45 (20.2) | 15 (16.5) | 0.53 |
| Liver transplanted, N (%) | 8 (3.5) | 3 (3.1) | 1.00 |
| 4COL7S, ng/mL | 7.3± 2.6 | 5.2± 1.9 | <0.0001**** |
| AST, IU/L | 59.9± 44.5 | 36.5± 19.7 | <0.0001**** |
| ALT, IU/L | 57.8± 47.5 | 35.1± 28.2 | <0.0001**** |
| γGTP, IU/L | 34 [23-59.5] | 24 [16-38] | <0.0001**** |
| Platelet, $\times 10^4$ /μL | 15.2± 7.0 | 17.9± 5.9 | 0.0013** |
| FIB-4 index | 5.30± 7.53 | 3.10± 2.58 | 0.0069** |
| APRI | 1.75± 4.20 | 0.73± 0.68 | 0.0221* |
| Forns index | 7.59± 2.21 | 6.59± 1.90 | 0.0002*** |

Data are shown as mean ±SD or median with interquartile range in brackets, as appropriate.
† 4COL7S-defined fibrosis progression$_{SVR}$ or regression$_{SVR}$: a change rate of serum 4COL7S from pre-treatment to SVR12≥ 0% is defined as fibrosis progression$_{SVR}$; and < 0% is defined as fibrosis regression$_{SVR}$.
Abbreviations: SOF, sofosbuvir; DAA, direct antiviral agents; HCC, hepatocellular carcinoma; 4COL7S, type IV collagen 7S fragment; AST, aspartate aminotransferase; ALT, alanine aminotransferase; γGTP, γ-glutamyl transpeptidase.

**S3 Table. Background parameters and clinical outcomes of study participants in exploratory analysis 1.**

| Parameters | Exploratory analysis 1 |
|---|---|
| N | 23 |
| Background parameters | |
| Sex (M/F), N (%) | 7 (30.4) / 16 (69.6) |
| Age, years | 70 ± 8 |
| SOF-based DAA, N (%) | 18 (78.3) |
| Prior history of HCC, N (%) | 3 (13.0) |
| Liver transplanted, N (%) | 1 (4.3) |
| Non-invasive fibrosis parameters | |
| Pre 4COL7S, ng/mL | 5.99 ± 1.5 |
| EOT 4COL7S, ng/mL | 5.56 ± 1.7 |
| SVR12 4COL7S, ng/mL | 5.20 ± 1.3 |
| Clinical Outcomes | |
| Newly emerged HCC post SVR12, N (%) | 2 (8.6) |
| All-cause mortality, N (%) | 1 (4.3) |

Data are shown as mean ±standard deviation.

Abbreviations: M, male; F, female; SOF, sofosbuvir; DAAs, direct antiviral agents; Pre, pre-treatment; 4COL7S, type IV collagen 7S fragment; EOT, end of treatment; HCC, hepatocellular carcinoma; SVR12, sustained virological response at week 12 after the end of treatment.

**S4 Table. Detailed background parameters of study subject for exploratory analysis 2**

| Progression/ Regression† | Sex | Age, years | SOF-based | Prior HCC | Survival | Post-SVR HCC | Pre FIB-4 | Pre eGFR§ | F/U, months | Pre 4COL7S | EOT 4COL7S |
|---|---|---|---|---|---|---|---|---|---|---|---|
| Progression | M | 80 | Y | N | Y | N | 1.77 | 53 | 27 | 4.7 | 5 |
| Progression | F | 82 | Y | Y | Y | N | 3.29 | 48 | 51 | 5.6 | 6.1 |
| Progression | F | 53 | N | N | Y | N | 1.87 | 87 | 45 | 4.2 | 4.8 |
| Progression | F | 40 | Y | N | Y | N | 1.20 | 67 | 50 | 4 | 4.6 |
| Progression | F | 48 | Y | N | Y | N | 1.18 | 54 | 54 | 4.2 | 4.4 |
| Progression | F | 74 | Y | N | Y | N | 2.25 | 63 | 48 | 4.2 | 5.6 |
| Progression | F | 82 | Y | Y | Y | Y | 6.78 | 54 | 28 | 7.0 | 7.6 |
| Regression | F | 60 | Y | N | Y | N | 2.15 | 82 | 32 | 8.4 | 7.4 |
| Regression | F | 60 | Y | N | Y | N | 4.98 | 75 | 15 | 6.4 | 4.9 |
| Regression | F | 74 | Y | N | Y | N | 4.42 | 67 | 52 | 6.3 | 6 |
| Regression | M | 67 | Y | N | Y | N | 2.70 | 59 | 48 | 4.7 | 4.1 |
| Regression | M | 73 | Y | N | Y | N | 1.61 | 65 | 52 | 4.9 | 4.3 |
| Regression | F | 42 | Y | N | Y | N | 1.35 | 70 | 49 | 5.3 | 4.3 |
| Regression | F | 56 | Y | N | Y | N | 3.26 | 63 | 49 | 5.3 | 4.8 |
| Statistics (P) | 1.00 | 0.56 | 1.00 | 0.46 | 1.00 | 1.00 | 0.64 | 0.20 | 0.95 | 0.12 | 0.60 |

† 4COL7S-defined fibrosis progression or regression: a change rate of serum 4COL7S from pre-treatment to EOT≥ 0% is defined as progression, and < 0% is defined as regression.

§ Unit: mL/min.1.73m$^2$.

Abbreviations: SOF, sofosbuvir; HCC, hepatocellular carcinoma; 4COL7S, type IV collagen 7S fragment; eGFR, estimated glomerular filtration rate; Pre, pre-treatment; EOT, end of treatment; SVR, sustained virological response.

**S5 Table. Datasets used for analysis (excel file)**
