# Supplementary material for: Dynamics of type IV collagen 7S fragment on eradication of HCV with direct antiviral agents: Prognostic and metabolomic impacts
Source: PLoS One. 2022 Oct 27;17(10):e0276925. doi: 10.1371/journal.pone.0276925 (PMC9612469; doi:10.1371/journal.pone.0276925)
Supplement: S1 Checklist — (DOC) [file pone.0276925.s003.doc]

STROBE Statement—Checklist of items that should be included in reports of ***cohort studies***

|  | Item No | Recommendation | Page/Lines |
| --- | --- | --- | --- |
| **Title and abstract** | 1 | (*a*) Indicate the study’s design with a commonly used term in the title or the abstract | Lines 65-72, Page 4 |
|  |  | (*b*) Provide in the abstract an informative and balanced summary of what was done and what was found | Lines 73-89, Pages 4-5 |
| Introduction | | |  |
| Background/rationale | 2 | Explain the scientific background and rationale for the investigation being reported | Lines 97-145, Pages 6-7 |
| Objectives | 3 | State specific objectives, including any prespecified hypotheses | Lines 62-63, Page 4; Lines 97-145, Page 6-7 |
| Methods | | |  |
| Study design | 4 | Present key elements of study design early in the paper | Lines 160-194, Pages 8-9 |
| Setting | 5 | Describe the setting, locations, and relevant dates, including periods of recruitment, exposure, follow-up, and data collection | Lines 150-183, Pages 8-9 |
| Participants | 6 | (*a*) Give the eligibility criteria, and the sources and methods of selection of participants. Describe methods of follow-up | Lines 156-183, Page 8-9 |
|  |  | (*b*) For matched studies, give matching criteria and number of exposed and unexposed | Lines 210-213, Page 10 (exploratory nested case-control analysis 2) |
| Variables | 7 | Clearly define all outcomes, exposures, predictors, potential confounders, and effect modifiers. Give diagnostic criteria, if applicable | Lines 163-183, Page 8-9 |
| Data sources/ measurement | 8* | For each variable of interest, give sources of data and details of methods of assessment (measurement). Describe comparability of assessment methods if there is more than one group | Lines 195-207, Page 10 |
| Bias | 9 | Describe any efforts to address potential sources of bias | Lines 565-578, Page 23 |
| Study size | 10 | Explain how the study size was arrived at | Not applicable due to the explorative nature of this study. |
| Quantitative variables | 11 | Explain how quantitative variables were handled in the analyses. If applicable, describe which groupings were chosen and why | Lines 287-295, Page 13 |
| Statistical methods | 12 | (*a*) Describe all statistical methods, including those used to control for confounding | Lines 243-266, Pages 11-12 |
|  |  | (*b*) Describe any methods used to examine subgroups and interactions | Lines 255-257, Page 12 |
|  |  | (*c*) Explain how missing data were addressed | Not applicable |
|  |  | (*d*) If applicable, explain how loss to follow-up was addressed | Not applicable |
|  |  | (*e*) Describe any sensitivity analyses | Not applicable |
| Results | | |  |
| Participants | 13* | (a) Report numbers of individuals at each stage of study—eg numbers potentially eligible, examined for eligibility, confirmed eligible, included in the study, completing follow-up, and analysed | S1 Fig; Lines 271-286, Page 13 |
|  |  | (b) Give reasons for non-participation at each stage | Lines 166-168, Page 8 |
|  |  | (c) Consider use of a flow diagram | S1 Fig |
| Descriptive data | 14* | (a) Give characteristics of study participants (eg demographic, clinical, social) and information on exposures and potential confounders | Lines 271-286, Pages 13-14; Table 1 |
|  |  | (b) Indicate number of participants with missing data for each variable of interest | Not applicable |
|  |  | (c) Summarise follow-up time (eg, average and total amount) | Line 271, Page 13 |
| Outcome data | 15* | Report numbers of outcome events or summary measures over time | Lines 281-286, Page 13 |
| Main results | 16 | (*a*) Give unadjusted estimates and, if applicable, confounder-adjusted estimates and their precision (eg, 95% confidence interval). Make clear which confounders were adjusted for and why they were included | Table 2 |
|  |  | (*b*) Report category boundaries when continuous variables were categorized | Lines 287-311, Pages 13-14 |
|  |  | (*c*) If relevant, consider translating estimates of relative risk into absolute risk for a meaningful time period | Not applicable |
| Other analyses | 17 | Report other analyses done—eg analyses of subgroups and interactions, and sensitivity analyses | Lines 378-462, Pages16-19 |
| Discussion | | |  |
| Key results | 18 | Summarise key results with reference to study objectives | Lines 478-488, Page 20 |
| Limitations | 19 | Discuss limitations of the study, taking into account sources of potential bias or imprecision. Discuss both direction and magnitude of any potential bias | Lines 565-578, Page 23 |
| Interpretation | 20 | Give a cautious overall interpretation of results considering objectives, limitations, multiplicity of analyses, results from similar studies, and other relevant evidence | Lines 489-584, Pages 21-23 |
| Generalisability | 21 | Discuss the generalisability (external validity) of the study results | Not applicable |
| Other information | | |  |
| Funding | 22 | Give the source of funding and the role of the funders for the present study and, if applicable, for the original study on which the present article is based | (Funding information: within the submission system) |

*Give information separately for exposed and unexposed groups.

**Note:** An Explanation and Elaboration article discusses each checklist item and gives methodological background and published examples of transparent reporting. The STROBE checklist is best used in conjunction with this article (freely available on the Web sites of PLoS Medicine at http://www.plosmedicine.org/, Annals of Internal Medicine at http://www.annals.org/, and Epidemiology at http://www.epidem.com/). Information on the STROBE Initiative is available at http://www.strobe-statement.org.
