# Supplementary material for: Dynamics of type IV collagen 7S fragment on eradication of HCV with direct antiviral agents: Prognostic and metabolomic impacts
Source: PLoS One. 2022 Oct 27;17(10):e0276925. doi: 10.1371/journal.pone.0276925 (PMC9612469; doi:10.1371/journal.pone.0276925)
Supplement: Attachment — Submitted filename: Point_by_point_reply.docx [file pone.0276925.s004.docx]

*PLOS ONE*

Dear Associate Editor,

***‘Dynamics of type IV collagen 7S fragment on eradication of HCV with direct antiviral agents: prognostic and metabolomic impacts’***

We are pleased to submit our revised manuscript for consideration for publication in the journal.

We have replied to all the insightful and valuable comments and have revised accordingly to the reviewers’ requests. Please refer to the revised manuscript (with and without tracked changes), the point-by-point reply, a revised S1A Fig, and a new Supporting Table 5 (excel file) containing all the dataset needed for analysis.

According to the instructions for publication requirements, we are glad to make the following statements.

-Funding statements:

This study was supported by grants-in-aid from the Keio Gijuku Academic Development Funds and the Mitsubishi Tanabe Pharma Corporation. The funders had no role in study design, data collection and analysis, decision to publish, or preparation of the manuscript. There was no additional external funding received for this study.

-Competing interests:

Y.K. is an employee at Mitsubishi Tanabe Pharma Corporation. The remaining authors declare no competing interests. This does not alter our adherence to PLOS ONE policies on sharing data and materials.

Thank you again for your kind consideration. We look forward to learning the result of the peer reviews in due course.

Sincerely yours,

Po-sung Chu, MD, PhD and Nobuhiro Nakamoto, MD, PhD,

Corresponding Authors

Department of Gastroenterology and Hepatology,

Division of Internal Medicine, School of Medicine, Keio University,

35 Shinanomachi, Shinjuku-ku

Tokyo 160-8582, Japan

Tel: +81-3-3353-1211; Fax: +81-3-3353-6247

E-mail: [pschu0928@iCloud.com](mailto:pschu0928@iCloud.com) and [nobuhiro@z2.keio.jp](mailto:nobuhiro@z2.keio.jp)

**Point-by-point Reply (PONE-D-22-21128.R1)**

Dynamics of type IV collagen 7S fragment on eradication of HCV with direct antiviral agents: prognostic and metabolomic impacts

PLOS ONE

Dear Dr. Chu,

Thank you for submitting your manuscript to PLOS ONE. After careful consideration, we feel that it has merit but does not fully meet PLOS ONE’s publication criteria as it currently stands. Therefore, we invite you to submit a revised version of the manuscript that addresses the points raised during the review process.

==============================

In the discussion, it needs to add a more explicit explanation about the underlying mechanisms to the current findings to improve the scientific merits which is a critical criterion for future acceptance of the paper.

Please ensure that your decision is justified on PLOS ONE’s [publication criteria](https://journals.plos.org/plosone/s/criteria-for-publication) and not, for example, on novelty or perceived impact.
==============================

**Reply:** Thank you so much for this valuable comment and this chance for revision. We have answered all the comments from the reviewers and have explicitly revised our explanations about the underlying mechanisms in the Discussion section as shown below. To further meet the publication criteria of this Journal, we have added a short paragraph summarizing the results at the beginning of the Discussion section (**Lines 476-479**), some short opening texts for each subsection of Discussion (**Lines 481-482; Lines 522-523**), and we moved the part of the discussion regarding exploratory analysis 1 to the last part of the discussion regarding the associations between non-invasive fibrosis markers and ECM remodeling (**Lines 534-548**). Thank you.

Please submit your revised manuscript by October 13, 2022. If you will need more time than this to complete your revisions, please reply to this message or contact the journal office at [plosone@plos.org](mailto:plosone@plos.org). When you're ready to submit your revision, log on to <https://www.editorialmanager.com/pone/> and select the 'Submissions Needing Revision' folder to locate your manuscript file.

Please include the following items when submitting your revised manuscript:

- A rebuttal letter that responds to each point raised by the academic editor and reviewer(s). You should upload this letter as a separate file labeled 'Response to Reviewers'.
- A marked-up copy of your manuscript that highlights changes made to the original version. You should upload this as a separate file labeled 'Revised Manuscript with Track Changes'.
- An unmarked version of your revised paper without tracked changes. You should upload this as a separate file labeled 'Manuscript'.

If you would like to make changes to your financial disclosure, please include your updated statement in your cover letter. Guidelines for resubmitting your figure files are available below the reviewer comments at the end of this letter.

If applicable, we recommend that you deposit your laboratory protocols in [protocols.io](http://protocols.io) to enhance the reproducibility of your results. [Protocols.io](http://Protocols.io) assigns your protocol its own identifier (DOI) so that it can be cited independently in the future. For instructions see: <https://journals.plos.org/plosone/s/submission-guidelines#loc-laboratory-protocols>. Additionally, PLOS ONE offers an option for publishing peer-reviewed Lab Protocol articles, which describe protocols hosted on [protocols.io](http://protocols.io). Read more information on sharing protocols at <https://plos.org/protocols?utm_medium=editorial-email&utm_source=authorletters&utm_campaign=protocols>.

We look forward to receiving your revised manuscript.

Kind regards,

Youkyung H. Choi, Ph.D.
Academic Editor
PLOS ONE

Journal Requirements:

When submitting your revision, we need you to address these additional requirements.

1. Please ensure that your manuscript meets PLOS ONE's style requirements, including those for file naming. The PLOS ONE style templates can be found at 
<https://journals.plos.org/plosone/s/file?id=wjVg/PLOSOne_formatting_sample_main_body.pdf> and 
<https://journals.plos.org/plosone/s/file?id=ba62/PLOSOne_formatting_sample_title_authors_affiliations.pdf>.

**Reply:** Thank you. We have confirmed this.

2. You indicated that you had ethical approval for your study. Please clarify whether minors (patients below the age of 18 years) were included in your study. If yes, in your Methods section, please ensure you have also stated whether you obtained consent from parents or guardians of the minors included in the study or whether the research ethics committee or IRB specifically waived the need for their consent.

**Reply:** We have added a statement clarifying that no study subjects aged below 18 years were included (**Lines 156-157**).

3. Thank you for stating in your Funding Statement: 
"This study was supported in part by grants-in-aid from the Keio Gijuku Academic Development Funds and the Mitsubishi Tanabe Pharma Corporation. The funders had no role in study design, data collection and analysis, decision to publish, or preparation of the manuscript".

Please provide an amended statement that declares *all* the funding or sources of support (whether external or internal to your organization) received during this study, as detailed online in our guide for authors at <http://journals.plos.org/plosone/s/submit-now>.  Please also include the statement “There was no additional external funding received for this study.” in your updated Funding Statement. 
Please include your amended Funding Statement within your cover letter. We will change the online submission form on your behalf.

**Reply:** We have amended this statement as “This study was supported by grants-in-aid from the Keio Gijuku Academic Development Funds and the Mitsubishi Tanabe Pharma Corporation. The funders had no role in study design, data collection and analysis, decision to publish, or preparation of the manuscript. There was no additional external funding received for this study.” We also included this statement in the cover letter. We apologize that the phrase “in part” was misleading, and we deleted this.

4. Thank you for stating the following in the Competing Interests section: 
"Y.K. is an employee at Mitsubishi Tanabe Pharma Corporation. The remaining authors declare no competing interests".

Please confirm that this does not alter your adherence to all PLOS ONE policies on sharing data and materials, by including the following statement: "This does not alter our adherence to PLOS ONE policies on sharing data and materials.” (as detailed online in our guide for authors <http://journals.plos.org/plosone/s/competing-interests>).  If there are restrictions on sharing of data and/or materials, please state these. Please note that we cannot proceed with consideration of your article until this information has been declared.

Please include your updated Competing Interests statement in your cover letter; we will change the online submission form on your behalf.

**Reply:** Thank you. We have confirmed that there are no restrictions on sharing data and/or materials. We have added the statement “Y.K. is an employee at Mitsubishi Tanabe Pharma Corporation. The remaining authors declare no competing interests. This does not alter our adherence to PLOS ONE policies on sharing data and materials.” We have also included this statement in the cover letter.

5. In your Data Availability statement, you have not specified where the minimal data set underlying the results described in your manuscript can be found. PLOS defines a study's minimal data set as the underlying data used to reach the conclusions drawn in the manuscript and any additional data required to replicate the reported study findings in their entirety. All PLOS journals require that the minimal data set be made fully available. For more information about our data policy, please see <http://journals.plos.org/plosone/s/data-availability>.

Upon re-submitting your revised manuscript, please upload your study’s minimal underlying data set as either Supporting Information files or to a stable, public repository and include the relevant URLs, DOIs, or accession numbers within your revised cover letter. For a list of acceptable repositories, please see <http://journals.plos.org/plosone/s/data-availability#loc-recommended-repositories>. Any potentially identifying patient information must be fully anonymized.

Important: If there are ethical or legal restrictions to sharing your data publicly, please explain these restrictions in detail. Please see our guidelines for more information on what we consider unacceptable restrictions to publicly sharing data: <http://journals.plos.org/plosone/s/data-availability#loc-unacceptable-data-access-restrictions>. Note that it is not acceptable for the authors to be the sole named individuals responsible for ensuring data access.

We will update your Data Availability statement to reflect the information you provide in your cover letter.

**Reply:** We have created a new **Supporting Table S5** (**and Line 811**) as an Excel file that includes all the datasets used for analysis in this study. Thank you.

Additional Editor Comments:

Dear Dr. Chu,

Thank you for submitting your manuscript to PLOS ONE. After careful consideration, we feel that it has merit, but is not suitable for publication as it currently stands. Therefore, my decision is "Major Revision."
We invite you to submit a revised version of the manuscript that addresses all points below by the reviewers. Particularly, a more explicit explanation about the underlying mechanisms to the current findings to improve the scientific merits which is a critical criterion for future acceptance of the paper.

**Reply:** Thank you so much for this valuable comment and this chance for revision. We have answered all the comments from the reviewers and have explicitly revised our explanations about the underlying mechanisms in the Discussion section as shown below. To further meet the publication criteria of this Journal, we have added a short paragraph summarizing the results at the beginning of the Discussion section (**Lines 476-479**), some short opening texts for each subsection of Discussion (**Lines 481-482; Lines 522-523**), and we moved the part of the discussion regarding exploratory analysis 1 to the last part of the discussion regarding the associations between non-invasive fibrosis markers and ECM remodeling (**Lines 534-548**). Thank you.

[Note: HTML markup is below. Please do not edit.]

Reviewers' comments:
Reviewer's Responses to Questions

**Comments to the Author**
1. Is the manuscript technically sound, and do the data support the conclusions?
The manuscript must describe a technically sound piece of scientific research with data that supports the conclusions. Experiments must have been conducted rigorously, with appropriate controls, replication, and sample sizes. The conclusions must be drawn appropriately based on the data presented.

Reviewer #1: Yes

Reviewer #2: Yes

2. Has the statistical analysis been performed appropriately and rigorously?

Reviewer #1: Yes

Reviewer #2: Yes

3. Have the authors made all data underlying the findings in their manuscript fully available?

The [PLOS Data policy](http://www.plosone.org/static/policies.action#sharing) requires authors to make all data underlying the findings described in their manuscript fully available without restriction, with rare exception (please refer to the Data Availability Statement in the manuscript PDF file). The data should be provided as part of the manuscript or its supporting information, or deposited to a public repository. For example, in addition to summary statistics, the data points behind means, medians and variance measures should be available. If there are restrictions on publicly sharing data—e.g. participant privacy or use of data from a third party—those must be specified.

Reviewer #1: Yes

Reviewer #2: Yes

4. Is the manuscript presented in an intelligible fashion and written in standard English?

PLOS ONE does not copyedit accepted manuscripts, so the language in submitted articles must be clear, correct, and unambiguous. Any typographical or grammatical errors should be corrected at revision, so please note any specific errors here.

Reviewer #1: Yes

Reviewer #2: Yes

5. Review Comments to the Author
Please use the space provided to explain your answers to the questions above. You may also include additional comments for the author, including concerns about dual publication, research ethics, or publication ethics. (Please upload your review as an attachment if it exceeds 20,000 characters)

Reviewer #1: The authors assessed the dynamics of 4COL7S in HCV patients who attained SVR with DAAs and correlated the trends of 4COL7S with the all-cause mortality and liver-related mortality. Furthermore, they also performed exploratory studies to prove the association of fibrinolysis/fibrogenesis markers and metabolomic profiles with 4COL7S dynamics to corroborate the systemic and intra-hepatic change of biomarkers. The manuscript is interesting and can provide insight as the clinical utility for the outcome prediction in HCV-SVR patients.

1. Supplementary Figure 1: The range of symbol of changes of 4COL7S should be extended from EOT to SVR12 because the authors stated that the changes were calculated by 4COL7S values at SVR12 minus those at baseline in Table 1 footnotes. Please confirm the time interval of the 4COL7S-defined progression or regression in the current study (baseline to EOT or baseline to SVR12).

**Reply:** thank you so much for your comment. We apologize for this confusing study scheme presented in the original S1A Fig. In its revised form, we indicate clearly that the red arrow to be the duration of treatment in “Supporting Information” in the manuscript (**Lines 787-788**). We also clearly indicate with arrowheads from Pre (baseline) to SVR12 for definitions of 4COL7S-defined fibrosis dynamics. We also added arrowheads from Pre (baseline) to EOT used in the exploratory analyses. Please refer to the **revised S1A Fig_r1**.

2. Line 157: Please clearly state the kit information used to determined SVR (limit of detection, manufacturer, company, kits name, countries etc.…). Furthermore, please also provide the similar information about 4COL7S (line 196).

**Reply:** Thank you for this critical comment. We have added information regarding the kits used for the analyses. Please refer to **Lines 158-160** and **Lines 190-191** in the revised manuscript. Thank you.

3. Line 173: The description “Before, during, and after achieving SVR” was confusing, and please re-write this sentence.

**Reply**: Thank you. We have rewritten this sentence as “During treatment and follow-up” Please refer to **Lines 176** in the revised manuscript.

4. Line 183: Table 1 should be moved to the Results section. Furthermore, please trim the wordings in line 183.

**Reply:** Thank you for this comment. We have moved Table 1 to the first part of the Results section, as you suggested. We have also trimmed the original Line 183 and added **Line 269-270** in the revised manuscript.

5. Line 290: I guessed that cut-off value of 6.1 was chosen by Youden index, and please state the sensitivity, specificity, PPV and NPV to predict FIB-4 > 3.25 with this cut-off value.

**Reply:** Thank you for this critical comment. Indeed, as we describe in the Statistics section of the Methods, we used Youden index to find the cut-off value. We have added this information to **Lines 301-303** in the revised manuscript.

6. It is intriguing why patients with more severe hepatic fibrosis at baseline tended to have regression of 4COL7S, while those with milder ones had the opposite trend. Please explain and proposed the potential mechanism.

**Reply:** Thank you so much for this critical comment. In fact, we have to say that we are not very clear about the underlying mechanism. First, we noticed that the association between the percent of change in 4COL7S from baseline to SVR and the baseline FIB-4 index is low (R= -0.237), even though it is statistically significant (**Fig_rev A and B**). In addition, there were many overlaps between the groups. We have tried to discuss about why there are variations in the dynamics of serum 4COL7S in the Discussion section. First, we discuss that “extra-hepatic” diseases may modify its dynamics (**Lines 555-573**). Second, we provided metabolomic clues that the gut-liver axis or liver-kidney axis may differ fundamentally between patients with 4COL7S-defined progression or regression (**Lines 575-592**). However, further mechanistic investigations are needed to fully answer these questions.

7. It is also surprising to know that as high as 30% of patients who achieved SVR12 still had fibrosis progression based on the dynamics of 4COL7S, compared to the published reports using dynamic changes of FIB-4, transient elastography, etc… Could the authors have some explanation for this phenomenon?

**Reply:** Thank you for this insightful comment.

First of all, we noticed a careless miss in the abstract, and revised it to 29.8% in **Line 75**. We also added additional information on the number of patients (10, which stands for 3.1% of study subjects) whose change rates of 4COL7S were 0% (**Line 319**).

Regression of liver fibrosis has been observed in some, but not all, patients after HCV eradication (*Lee YA, et al. Gut. 2015; 64(5):830-41; Lee YA, and Friedman SL. Antiviral Res. 2014; 107:23-30*) Multiple previous reports have demonstrated that some patients with CHC might suffer from deteriorations of fibrosis after SVR; 9.5% of their FIB-4 index deteriorated (*Ioannou GN, et al. Gastroenterology. 2019; 157:1264-1278*); 12% of their liver fibrosis status deteriorated serologically (using FibroTest®; *Poynard T, et al. J Hepatol. 2013; 59(4):675-83*), 12% deteriorated histologically (assessed by percutaneous liver biopsy; *Maylin S, et al. Gastroenterology. 2008; 135(3):821-9*), 16.4% demonstrated worsened liver stiffness (assessed by transient elastography; *Sporea I et al. J Gastrointestin Liver Dis. 2017: 26(2):145-150*), and 31% demonstrated worsened intrahepatic α-smooth muscle actin, a marker of hepatic stellate cell activation, immunohistochemistry (D'Ambrosio R, et al. Hepatology. 2012; 56(2):532-43). Although the chronological effects (time after SVR) and background status of fibrosis are not homogenous in these studies, we think that what we have observed in this current study (29.8% of study subjects without a decrease in serum 4COL7S) is not too surprising. It is also interesting that non-invasive fibrosis tests comprising necro-inflammatory markers (such as AST or ALT in FIB-4) are prone to resolve (less percentage of deterioration) after SVR; however, markers of stellate cell activity (such as αSMA) show a lower tendency to resolve. We have to say that the real mechanism is still unclear, the dynamics of serum 4COL7S might reflect ECM remodeling activity, both in the liver and in other extrahepatic organs, during the process of HCV eradication.

8. Although 4COL7S, MMP and TIMP are indicators of fibrogenesis and fibrinolysis, they are not specific for the liver. While patients with HCV who achieve SVR may reduce intrahepatic inflammation which favors fibrinolysis, viral eradication also reduces the risk of systemic inflammation that also favors reduction of systemic fibrogenesis, with which noninvasive fibrosis markers are expected to be improved. That means as I proposed in Q6 and Q7 as how the authors explain a high percentage of progression by 4COL7S dynamics in SVR patients.

**Reply:** Thank you so much for this valuable viewpoint. We totally agree with you. As we discussed in point 7, various serological markers may have different dynamics in response to reduced inflammation, to viral eradication, or to ECM remodeling in the liver or other non-liver organs. We added a paragraph to the Discussion section as **Lines 525-526**. Thank you so much again for your insightful comment.

9. Lines 332 and 340: The authors stated that a baseline 4COL7S > 6.1 had the similar trend of all-cause or liver-related mortality and HCC with regression or progression of 4COL7S to overall population. How about the trends in patients with a baseline 4COL7S < 6.1? It would be important because the similar or different trends in patients with > 6.1 or > 6.1 may imply that the baseline advanced hepatic fibrosis would be a disease modifier or just a confounder.

**Reply:** Thank you for this critical comment. We have re-analyzed and plotted new figures for this point-by-point reply. For study subjects whose baseline 4COL7S was < 6.1, the Kaplan-Meier analysis for all-cause mortality (**Fig_rev C**) and that for newly emerged HCC (**Fig_rev D**) with stratification of 4COL7-defined fibrosis progression/regression (from baseline to SVR) are shown. Because there was only one event in the all-cause mortality analysis, it is difficult to draw any meaningful conclusions. For newly emerged HCC, it was similar to that in patients whose baseline 4COL7S were over 6.1, with no significant difference between regression/progression (Fig 2B and 2D). We believe that because mortality or hepatocarcinogenesis are more statistically related to advanced fibrosis at baseline (as we shown in S2G-H Fig), the number of patients whose baseline 4COL7S was < 6.1 was not large enough to draw a conclusion. We have added this point to the limitation (**Lines 597-601**). In addition, as stated in point 6, we noticed a significant but low-grade association between the percent of change in 4COL7S from baseline to SVR and baseline FIB-4 index (please refer to **Fig_rev A and B** in point 6). Because this low-grade association, we believe that it is difficult to say that the dynamics of 4COL7S are merely confounders of baseline fibrosis.

10. Line 491: “640 HCV-coinfected patients with CHC” should be “HIV-coinfected”.

**Reply:** We have corrected as you suggested (**Line 508**). We apologize for this careless error.

Reviewer #2: The goal of this study is to find non-invasive fibrosis markers and study their capability to predict post-SVR prognosis in patients. They analyzed the predictive value of Type IV collagen 7S after SVR in addition to trying to identify novel targets that can serve as non-invasive markers for liver fibrosis and post-SVR prognosis in patients.

Major suggestions:
1. The test used for serum 4COL7S level measurement should be more specifically described in the materials and methods. Since kits are commercially available full information about the kits should be provided. Is this kit clinically used specifically for liver fibrosis or is it approved for another disease? Are there any scientific studies that previously evaluated this kit? They should be cited. (lines 196-199)

**Reply:** Thank you so much for this important comment. We have added the detailed information about the commercially available kit for measuring serum 4COL7S (**Lines 190-191**). In Japan, the indication for measuring serum 4COL7S is only approved for patients with suspected chronic liver diseases. Unfortunately, we cannot find any previous report specified the kit from *Fujirebio Inc*., however. Because there is only one commercially available and approved kit for measuring 4COL7S in Japan, we added three previous reports that applied serum 4COL7S levels derived in clinical settings in Japan (**Lines 192-195**; new **refs 21-23**). In fact, ref 21 is reported previously by our group. We are interested in the possible role of any fibrosis marker inclusive of 4COL7S that may predict prognosis in a patient with any liver disease.

2. In the metabolome section, the filtrate was evaporated and then reconstituted (lines 221-223). Why was this step required? Were the samples stored and transported in this state?

**Reply:** Thank you so much for this critical comment. First, the samples were stored at -80℃ from collection. They were sent for analysis under the same conditions until the start of the analysis. We have revised the description of sample handling before analysis in **Line 212** in the Methods section. Second, we contacted the technician at *Human Metabolome Technologies Inc.* and confirmed why the steps of evaporation and reconstitution are needed. There are two purposes. One is for increased concentration (by decreasing volumes from 300μL to 50 μL) of metabolites may help increase the possibility of passing the lowest thresholds of detection/quantification, thereby improving the sensitivity of detection by chromatography. The other purpose is that reconstitution with 100% pure water (Milli-Q) may help increase the solubility of hydrophilic ionic metabolites.

3. The utility of the 4COL7S test should be better described by the authors. Some of the questions that should be answered are: a) Is there any value in using this test for prognosis of a single patient? b) What if this patient did not have this test done before the treatment? Is there still a use for it if tested post SVR? c) Although a difference between the groups is statistically significant, there is a large overlap between the groups. Is there a plan to study specificity and sensitivity of this test in predicting disease outcome?

**Reply:** Thank you so much for this insightful comment.

**(a)** As described in the reply to point 1, We have been interested in the possible role of any fibrosis marker inclusive of 4COL7S that may predict prognosis in a patient with any liver disease. In our previous report, serum levels of 4COL7S were prognostic in patients with non-acetaminophen associated acute liver failure (**ref 21***; 10.1002/hep4.1233*). In the current manuscript, we have also mentioned about several previous reports regarding 4COL7S for predicting fibrosis levels in chronic liver diseases (**refs 18-19, refs 22-23**) and non-hepatic diseases (new **refs 43-46**). Most of these are related to ECM remodeling. Except for ref 21 and the current study, none of them reported any correlations with the prognosis of a patient.

**(b)** In this current study, we focus on the “increase” or “decrease” of 4COL7S, however, as an “exact value” of 4COL7S, especially after SVR, cannot be determined by this current study, due to a relatively small number of study subjects. As we mentioned in the Discussion (**Lines 527-531**), the current international guidelines (EASL) suggest that “*thresholds for LSM and NITs used in untreated viral hepatitis have proven inaccurate after SVR and it is necessary to validate the newer (lower) cut-offs in larger studies*.” (**ref 38**= *EASL, 2021, DOI: 10.1016/j.jhep.2021.05.025*) It is also insightful to know that in a detailed analysis as we referred as new **ref 42**, a large cohort study with 48,123 patients (new **ref 42**= *Ioannou et al. Gastroenterology 2019, DOI: 10.1053/j.gastro.2019.07.033*), the authors used the trend of FIB-4 at the same cut-off value (3.25) for prognostic analysis. The concept of our current study was quite similar to that of a previous report by *Ioannou et al* (new **ref 42**). We have to say that our results are not applicable when only a single value of serum 4COL7S is available after SVR. In addition, we have made a clearer description when we refer to new **ref 42** in the Discussion section (**Line 551-553**).

**(c)** Indeed, if we look at every single value of “Δ% 4COL7S_SVR-Pre_” in this cohort, there is a large overlap between patients who died (all-cause; median= -12.2%, IQR= -24.0% ~ +2.0%) and those who did not (median= +1.4%, IQR= -7.4% ~ +13.4%) during observation (data not shown in the manuscript). However, we used Kaplan-Meier analysis (**Fig 2**) and Cox proportional hazard analysis (**Table 2**) to overcome possible bias due to various observational periods, rather than finding a single cut-off value for prognosis prediction. In fact, if we set Δ% 4COL7S_SVR-Pre_= 0 as a cut-off, the sensitivity of predicting all-cause mortality was 60.0% and the specificity was 71.2%. We believe that a larger number and a longer observational periods in the future should refine diagnostic accuracy of a single cut-off value.

4. The metabolomic analysis is based on the 4COL7S-defined fibrosis. Why was it not based on the currently used clinical evaluation of the patients.

**Reply:** Thank you so much for this critical comment. As we replied in point 3 (b), and as we mentioned in Discussion (**Lines 527-531**), the current international guidelines (EASL) suggest that “*thresholds for LSM and NITs used in untreated viral hepatitis have proven inaccurate after SVR and it is necessary to validate the newer (lower) cut-offs in larger studies*” (**ref 38**= *EASL, 2021, DOI: 10.1016/j.jhep.2021.05.025*) In other words, even the most frequently applied LSM, a generally acceptable threshold is not yet available. In our current study, our observation derived from 4COL7S has shown prognostic values in the first part (**Fig 2**), so we decided to use 4COL7S to define fibrosis dynamics in the metabolomic analysis. In other words, 4COL7S itself just simply connects the story that we aimed to tell.

5. What are the plans for the metabolomic analysis results? What was the purpose of performing this experiment, and how will these results be used in the future.

**Reply:** Thank you for this important comment. The purpose of performing metabolomic analysis was stated in **Lines 423-424**. Since we observed that serum 4COL7S levels did not improve in 29.8% of our study subjects and that they suffered from a higher risk of all-cause mortality after SVR, we want to know if there is any correlation between metabolomic differences and 4COL7S dynamics. HCV infects hepatocytes, the main cells that control metabolism, which is another reason for us to perform metabolomic analysis. As we stated in the Results section (**Lines 441-456**) and the Discussion section (**Lines 579-592**), these results helped us to know the dynamics of 4COL7S after SVR may be related to the gut-liver axis or the liver-kidney associations. We also hope that the results will help target any specific metabolite or pathway to manipulate the dynamics of 4COL7S (possibly through hepatic stellate cells or fibrinolytic macrophages) in the future. Because the latter part of our future plan is quite beyond the scope of this research, we did not include this part in the Discussion section.

Minor suggestions:
1. This manuscript uses a lot of abbreviations, and although they are all defined in the beginning of the manuscript, it gets difficult to follow and clearly understand some parts of this manuscript.

**Reply:** Thank you for this comment. We have done our best to clarify our manuscript by listing all the abbreviations and using full spelling at the first appearance. Since there are many main parts knowledge entities in this study, such as clinical virology, pharmacotherapeutics of HCV treatment, fibrosis/cirrhosis, ECM remodeling, and metabolism, etc., we find that it is impossible to reduce the frequency of using abbreviations. We apologize for this point.

2. In addition, readability may be improved by having a native English speaker edit the manuscript.

**Reply:** Thank you. We had the manuscript and this point-by-point reply edited by a native English speaker (*Editage©*). Changes due to original grammar flaws are marked with red color, otherwise specifically mentioned. We hope that this has improved the readability of this revised manuscript.

6. PLOS authors have the option to publish the peer review history of their article ([what does this mean?](https://journals.plos.org/plosone/s/editorial-and-peer-review-process#loc-peer-review-history)). If published, this will include your full peer review and any attached files.


If you choose “no”, your identity will remain anonymous but your review may still be made public.


**Do you want your identity to be public for this peer review?** For information about this choice, including consent withdrawal, please see our [Privacy Policy](https://www.plos.org/privacy-policy).

Reviewer #1: No

Reviewer #2: No

[NOTE: If reviewer comments were submitted as an attachment file, they will be attached to this email and accessible via the submission site. Please log into your account, locate the manuscript record, and check for the action link "View Attachments". If this link does not appear, there are no attachment files.]

While revising your submission, please upload your figure files to the Preflight Analysis and Conversion Engine (PACE) digital diagnostic tool, <https://pacev2.apexcovantage.com/>. PACE helps ensure that figures meet PLOS requirements. To use PACE, you must first register as a user. Registration is free. Then, login and navigate to the UPLOAD tab, where you will find detailed instructions on how to use the tool. If you encounter any issues or have any questions when using PACE, please email PLOS at [figures@plos.org](mailto:figures@plos.org). Please note that Supporting Information files do not need this step.
